# Supplementary material for: Construction of Enzyme-Responsive Micelles Based on Theranostic Zwitterionic Conjugated Bottlebrush Copolymers with Brush-on-Brush Architecture for Cell Imaging and Anticancer Drug Delivery
Source: Molecules. 2022 May 7;27(9):3016. doi: 10.3390/molecules27093016 (PMC9101325; doi:10.3390/molecules27093016)
Supplement: Supplementary file 1 [file molecules-27-03016-s001.zip › molecules-1692514-supplementary.pdf]

*Supporting Information*

# Construction of Enzyme-Responsive Micelles Based on Theranostic Zwitterionic Conjugated Bottlebrush Copolymers with Brush-on-Brush Architecture for Cell Imaging and Anticancer Drug Delivery

**Fangjun Liu [2], Dun Wang [1], Jiaqi Wang [1], Liwei Ma [2], Cuiyun Yu [1,*] and Hua Wei [1,2,*]**

[1] Hunan Province Cooperative Innovation Center for Molecular Target New Drug Study & Department of Pharmacy and Pharmacology, University of South China, Hengyang 421001, China; 20202012110818@stu.usc.edu.cn (D.W.); jiaqiwang@stu.usc.edu.cn (J.W.)
[2] State Key Laboratory of Applied Organic Chemistry, Key Laboratory of Nonferrous Metal Chemistry and Resources Utilization of Gansu Province, College of Chemistry and Chemical Engineering, Lanzhou University, Lanzhou, 730000, China; liufj18@lzu.edu.cn (F.L.); malw@lzu.edu.cn (L.M.)
* Correspondance: yucuiyunusc@hotmail.com (C.Y.); weih@usc.edu.cn (H.W.)

## Captions of Figures and Table

**Figure S1.** $^{1}H$ NMR spectra of PFONPN-*g*-$N_3$ in $CDCl_3$.

**Figure S2.** $^{1}H$ NMR spectra of alkyne-PHEMA, PFONPN-*g*-PHEMA and PFONPN-*g*-(PHEMA-*g*-$NH_2$) in DMSO-$d_6$.

**Table S1.** Experimental details for synthesis of polymer brushes

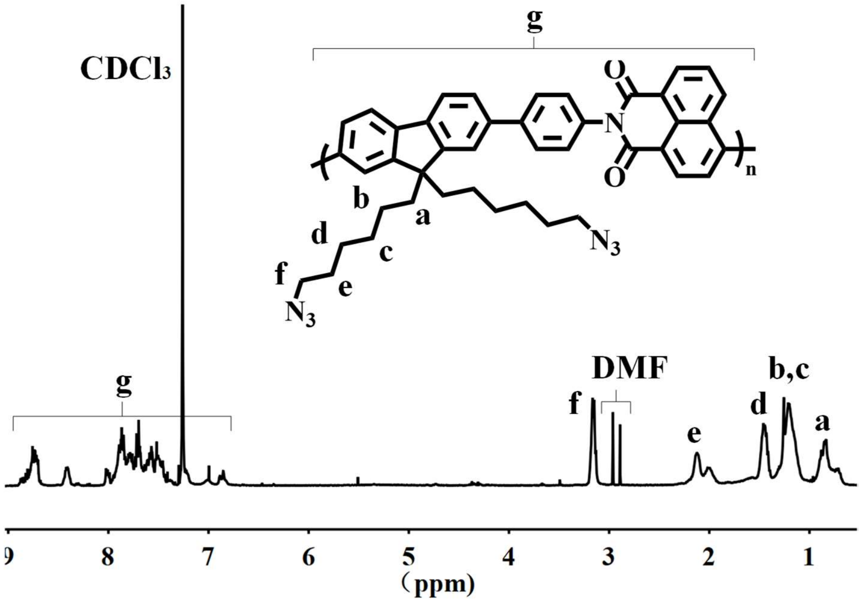


**Figure S1.** $^{1}$H NMR spectra of PFONPN-*g*-$N_3$ in $CDCl_3$.

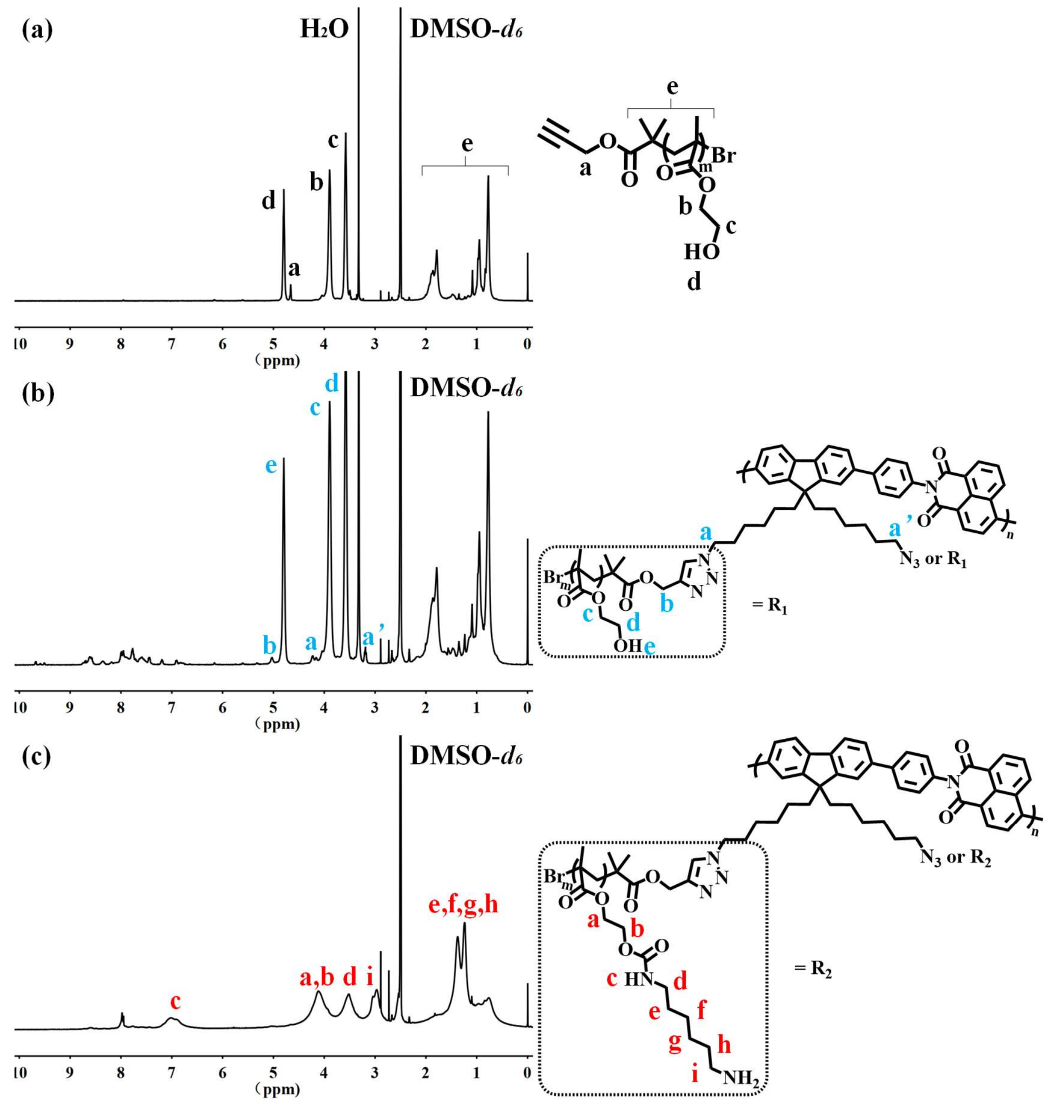


**Figure S2.** $^1H$ NMR spectra of alkyne-PHEMA, PFONPN-*g*-PHEMA and PFONPN-*g*-(PHEMA-*g*-$NH_2$) in DMSO-$d_6$.

**Table S1.** Experimental details for synthesis of polymer brushes

| Entry | [monomer]/[initiator] OEGMA/SBMA/ PFONPN$_9$-*g*-(PHEMA$_{15}$-*g*-(PTyr$_{16}$-Br$_2$)) | [monomer] [M] | DMSO:$H_2O$ /mL | Reaction time / h |
|---|---|---|---|---|
| $P_1$ | 30:0:1 | 0.06 | 10:0 | 1 |
| $P_2$ | 15:15:1 | 0.05 | 10:1 | 1 |
| $P_3$ | 60:0:1 | 0.12 | 10:0 | 1 |
| $P_4$ | 30:30:1 | 0.11 | 10:1 | 1 |
| $P_5$ | 70:30:1 | 0.18 | 10:1 | 1 |
